# Protection against diabetic cardiomyopathy is achieved using a combination of sulforaphane and zinc in type 1 diabetic OVE26 mice

**DOI:** 10.1111/jcmm.14520

**Published:** 2019-07-03

**Authors:** Jiqun Wang, Shudong Wang, Wanning Wang, Jing Chen, Zhiguo Zhang, Qi Zheng, Quan Liu, Lu Cai

**Affiliations:** ^1^ The Center of Cardiovascular Diseases The First Hospital of Jilin University Changchun China; ^2^ Pediatric Research Institute, Department of Pediatrics University of Louisville Louisville Kentucky USA; ^3^ Department of Nephrology The First Hospital of Jilin University Changchun China; ^4^ Department of Otolaryngology Stanford University Palo Alto California USA; ^5^ Department of Bioinformatics and Biostatistics University of Louisville Louisville Kentucky USA; ^6^ Departments of Radiation Oncology, Pharmacology and Toxicology University of Louisville Louisville Kentucky USA

**Keywords:** cardiomyopathy, diabetes, metallothionein, nuclear factor E2, related factor 2, sulforaphane, zinc

## Abstract

Sulforaphane (SFN) can effectively induce nuclear factor E2–related factor 2 (Nrf2), and zinc (Zn) can effectively induce metallothionein (MT), both of which have been shown to protect against diabetic cardiomyopathy (DCM). However, it is unclear whether combined treatment with SFN and Zn offers better cardiac protection than either one alone. Here, we treated 5‐week‐old OVE mice that spontaneously develop type 1 diabetes with SFN and/or Zn for 18 weeks. Cardiac dysfunction, by echocardiography, and pathological alterations and remodelling, shown by cardiac hypertrophy, fibrosis, inflammation and oxidative damage, examined by histopathology, Western blotting and real‐time PCR, were observed in OVE mice. All these dysfunction and pathological abnormalities seen in OVE mice were attenuated in OVE mice with treatment of either SFN, Zn or SFN/Zn, and the combined treatment with SFN/Zn was better than single treatments at ameliorating DCM. In addition, combined SFN and Zn treatment increased Nrf2 function and MT expression in the heart of OVE mice to a greater extent than SFN or Zn alone. This indicates that the dual activation of Nrf2 and MT by combined treatment with SFN and Zn may be more effective than monotherapy at preventing the development of DCM via complementary, additive mechanisms.

## 1 INTRODUCTION

Diabetes is one of the most common chronic diseases and is characterized by high blood glucose concentrations. The prevalence of diabetes and number of patients have continued to increase in recent years. In 2017, it was estimated that 425 million people aged 20‐79 years are living with diabetes, and the number of people with diabetes in this age group was predicted to rise to 629 million, representing 9.9% of the population, by 2045.^1^ 5%‐10% of global diabetes patients are type 1 diabetes (T1D),^2^ which is a multifactorial disease with a strong genetic component, caused by the autoimmune destruction of pancreatic β cells, culminating in absolute insulin deficiency.^3^, ^4^ Prevalence of T1D disease is increasing worldwide.^2^, ^5^, ^6^ Generally, longitudinal or cross‐sectional studies are often locally or regionally performed. Consequently, it is difficult to access generalizable results because the epidemiology of T1D is known to be heterogeneous regarding geography and ethnicity.^2^ Diabetic complications seriously affect human health and continue to be a major cause of morbidity and mortality in persons with T1D.^7^, ^8^ Cardiovascular disease events not only are more common in individuals with T1D as compared to those without diabetes, but also occur earlier.^9^ Diabetic cardiomyopathy (DCM) is the main cause of heart failure in diabetic patients and one of the most lethal complications.^10^, ^11^ DCM was originally reported by Rubler et al^12^ and is characterized by ventricular contractile dysfunction, cardiac hypertrophy, interstitial collagen accumulation and fibrosis.^13^, ^14^, ^15^, ^16^ The concept of DCM is often considered in patients who are specifically affected by type 2 diabetes (T2D); however, a cardiomyopathy, independent of hypertension, nephropathy or ischaemic heart disease induced by metabolism is also evident in patients with T1D.^17^ The mechanism of DCM is not fully understood, but oxidative stress is considered to be one of the most important pathophysiological factors.^18^, ^19^, ^20^ Therefore, treatments targeting oxidative stress may represent suitable strategies for the effective prevention of DCM.^21^, ^22^

Nuclear factor E2–related factor 2 (Nrf2), a transcription factor with high sensitivity to oxidative stress, binds to antioxidant response elements (AREs) in the nucleus and promotes the transcription of a wide variety of antioxidant genes. Nrf2 is located in the cytoskeleton, bound to Kelch‐like ECH‐associated protein 1 (Keap1).^23^ Oxidative stress causes Nrf2 to dissociate from Keap1 and to subsequently translocate into the nucleus, which results in its binding to AREs and the transcription of downstream target genes, including genes that encode antioxidants, detoxifying enzymes, antiapoptotic proteins and proteasomes,^24^, ^25^, ^26^ stress and prevent DCM.^27^, ^28^, ^29^, ^30^, ^31^

Metallothionein (MT) is a cysteine‐rich protein that can bind heavy metal ions such as copper and zinc (Zn).^32^ It is also a potent scavenger of free radicals because of its high thiol content.^33^ Therefore, as another effective antioxidant, MT may also be effective in protecting against DCM.^26^, ^34^, ^35^, ^36^, ^37^

Sulforaphane (SFN) is an isothiocyanate and is stored as its relatively stable precursor glucoraphanin (GRN) in a variety of cruciferous plants, with particularly high levels detected in broccoli and broccoli sprouts.^38^, ^39^, ^40^ When the plant is consumed, plant myrosinases or microbial hydrolases present in gut bacteria convert GRN to SFN.^41^ SFN can modify cysteine 151 in Keap1 to perturb the association of Cul3 ubiquitin ligase with Keap1, allowing Nrf2 to escape degradation by the proteasome. Thus, Nrf2 is stabilized and translocates into the nucleus to induce the transcription of its target genes.^39^ Many studies have shown that SFN has antioxidant, apoptosis‐inducing and anti‐inflammatory effects in diabetes, cardiovascular, neurologic and other diseases.^42^, ^43^, ^44^ Our previous studies have shown that MT is downstream of Nrf2^45^, ^46^ and regulates the expression of Nrf2 as well.^47^ We also found that Zn stimulates Nrf2 expression and transcription via activation of Akt‐dependent inhibition of Fyn nuclear translocation.^48^ Considering that SFN and Zn protective effects against diabetes‐induced organ damage rely on the activation of different molecular targets (Nrf2 and MT, respectively),^49^, ^50^, ^51^ and two pathways can interact with each other, we suggested that the combined treatment with SNF and Zn might provide better protection against diabetes‐induced complications than either treatment alone. However, published research has not determined whether this combination is more effective at relieving oxidative stress and preventing the development of DCM than either substance alone. Therefore, in this study, we used a genetic mouse model of T1D, the OVE26 mouse, to compare the effects of treatment with SFN, Zn or a combination of the two. The OVE26 mouse model has often been used to study of diabetes‐induced complications.^26^, ^52^ This is because OVE26 mice develop T1D within 24 hours after birth because of beta cell‐specific damage due to a calmodulin transgene regulated by the insulin promoter,^53^ and develop well‐characterized cardiac and renal complications. Additionally, this model avoids the potential systemic toxicity of streptozotocin, which is used to induce T1D in other animal models.^54^, ^55^, ^56^

## 2 MATERIALS AND METHODS

### 2.1 Animals

All animal procedures followed the NIH Guide for the Care and Use of Laboratory Animals and were approved by the University of Louisville Institutional Animal Care and Use Committee (IACUC) guidelines. Transgenic female OVE26 and FVB (wild‐type control) mice were maintained in a 12‐hour light/dark cycle at 25°C and given free access to water and food (10 kcal% fat with 30 mg Zn per 4057 kcal). OVE26 mice develop severe T1D within 24 hours of birth.^53^ We used female mice because cardiovascular disease is less likely in women owing to the protective effect of oestrogen. However, in the Framingham study, female patients with diabetes had a significantly higher risk of heart failure^57^; therefore, novel therapeutic approaches to diabetic complications developed in female models are likely to have greater clinical relevance.

### 2.2 In vivo administration of SFN and Zn

Mice were administered SFN (0.5 mg/kg; Sigma‐Aldrich) and/or zinc sulphate (5 mg/kg) by gavage from 5 weeks of age on 5 days per week for 18 weeks. The doses of SFN and Zn used were based on previous studies.^37^, ^58^ Vehicle control mice were administered an equivalent volume of phosphate‐buffered saline (PBS) containing 1% dimethyl sulphoxide. According to the conversion of animal doses to human equivalent doses based on body surface area guided by FDA,^59^ 5 mg/kg of Zn in mouse is only equivalent to 0.405 mg/kg in human being. This does not even exceed the upper limit of DRIs in a 90 kg human being. Similarly, 0.5 mg/kg of SFN in mouse is converted to human dose which is 0.0405 mg/kg. 300 g of broccoli contains about 2.63 mg of SFN,^60^ which may be higher after cooking for a short time.^61^ Therefore, the doses of Zn and SFN used in the present study are relatively low and safe. In addition, in many clinical studies, SFN and Zn were used to treat chronic diseases, and the doses of SFN and Zn were often higher than the doses used in the present study.^62^, ^63^, ^64^, ^65^, ^66^, ^67^ In our study, considering the intestinal malabsorption of OVE26 diabetic mice so that the absorption of Zn from normal diet may be reduced, we supplemented a little Zn besides diet, which is a nutritional treatment.

### 2.3 Echocardiography

To assess cardiac function, transthoracic echocardiography was performed on mice using a high‐resolution imaging system (Vevo 770; VisualSonics), as described previously.^68^ Briefly, mice were anaesthetized with isoflurane and placed in a supine position on a heating pad. Two‐dimensional and M‐mode echocardiography was used to assess wall motion, chamber dimensions and cardiac function.

### 2.4 Sirius Red staining

After anaesthesia, mouse hearts were isolated and fixed in 10% buffered formalin, and then dehydrated in a graded alcohol series, cleared with xylene, embedded in paraffin and sectioned at 5 μm thickness. Cardiac fibrosis was assessed following the staining of collagen fibres with 0.1% Sirius Red F3BA and 0.25% Fast Green FCF, as described previously.^34^

Tissue containing collagen was stained red, and myocardial tissue was stained green. ImageJ (National Institutes of Health) analysis of red areas was used to estimate collagen content.

### 2.5 Western blotting

Western blotting was performed as described previously.^69^ Briefly, heart tissue was homogenized in lysis buffer and proteins were collected by centrifugation at 12 000 r/min for 15 minutes at 4°C. Lysate protein concentration was determined using a Bradford assay. Samples of total protein were diluted in loading buffer, heated at 98°C for 5 minutes and then subjected to electrophoresis on 8% or 10% SDS‐PAGE gels. After electrophoresis, proteins were transferred to nitrocellulose membranes at 4°C, which were then rinsed briefly in Tris‐buffered saline, blocked in blocking buffer containing 5% milk and 0.5% bovine serum albumin for 1 hour, and then washed three times with Tris‐buffered saline containing 0.1% Tween‐20 (TBST). The membranes were incubated with primary antibodies overnight at 4°C, washed with TBST and then incubated with a secondary horseradish peroxidase‐conjugated antibody for 1 hour at room temperature. The antigen‐antibody complexes were then visualized using an ECL Kit (Bio‐Rad). MT expression was detected using a modified Western blotting protocol.^37^

The primary antibodies used in the study were as follows: 3‐nitrotyrosine (3‐NT; 1:1000; Millipore), 4‐hydroxynonenal (4‐HNE; 1:2000; Alpha Diagnostic International), plasminogen activator inhibitor type 1 (PAI‐1; 1:1000; BD Biosciences), haem oxygenase‐1 (HO‐1; 1:1000; Cell Signaling Technology), transforming growth factor beta (TGF‐β; 1:1000; Abcam), Nrf2 (1:1000; Abcam), fibronectin (1:1000; Abcam), collagen‐1 (1:1000; Abcam), tumour necrosis factor‐α (TNF‐α; 1:1000; Abcam), intercellular adhesion molecule‐1 (ICAM‐1; 1:1000; Santa Cruz Biotechnology), superoxide dismutase‐2 (SOD‐2; 1:5000; Santa Cruz Biotechnology), catalase (CAT; 1:5000; Santa Cruz Biotechnology), β‐actin (1:8000; Santa Cruz Biotechnology), glyceraldehyde 3‐phosphate dehydrogenase (GAPDH; 1:3000; Santa Cruz Biotechnology) and MT (1:1000; DakoCytomation).

Protein content was determined by measuring the grey value of the bands by Image Lab (Bio‐Rad, Hercules).

### 2.6 Quantitative real‐time PCR

Total RNA was extracted using TRIzol reagent (Invitrogen). RNA concentration and purity were assessed using a NanoDrop ND‐1000 spectrophotometer. First‐strand complementary DNA (cDNA) was synthesized from total RNA according to the manufacturer's protocol (Promega). Reverse transcription was performed with a Mastercycler Gradient (Eppendorf) at 42°C for 50 minutes, followed by 95°C for 5 minutes, with 0.5 μg total RNA in a final volume of 20 μL (4 μL of 25 mmol/L MgCl_2_, 4 μL AMV reverse transcriptase 5× buffer, 2 μL dNTPs, 0.5 μL RNase inhibitor, 1 μL AMV reverse transcriptase, 1 μL dT primer and nuclease‐free water). Primers targeting fibronectin, TGF‐β, MT1, CAT, SOD and GAPDH were purchased from Thermo Fisher. Quantitative PCR was performed in a 20 μL volume (10 μL TaqMan Universal PCR Master Mix, 1 μL primers and 9 μL cDNA) using the ABI 7300 Real‐Time PCR system. Data are expressed as fold differences vs controls, using the ΔΔCt method and GAPDH as a reference gene.

### 2.7 Statistical analysis

Data are expressed as the mean and standard deviation (SD) for each outcome variable. *T* test was performed to identify statistically significant differences between the means of two groups. One‐way analysis of variance (ANOVA) was performed, followed by the Tukey post hoc analysis to identify groups which are statistically significantly different. The normality assumption of *t* test and ANOVA was examined by the Shapiro‐Wilk test, and the equality of variance was validated by Levene's test. If the normality assumption was violated, a log transformation was then applied. We are fully aware of the small sample size in our study. However, the effect sizes are considerably large in the comparisons. Thus, even with a small size, the *t* test or one‐way ANOVA test has at least 80% power to detect a meaningful difference at a significance level at 0.05.^70^ The statistical analysis and graph preparation were undertaken using GraphPad Prism 7 (GraphPad Software Inc) and R (https://www.r-project.org/). Results were considered statistically significant when *P* < 0.05.

## 3 RESULTS

### 3.1 Characterization of DCM in T1D mice

**Figure 1:**
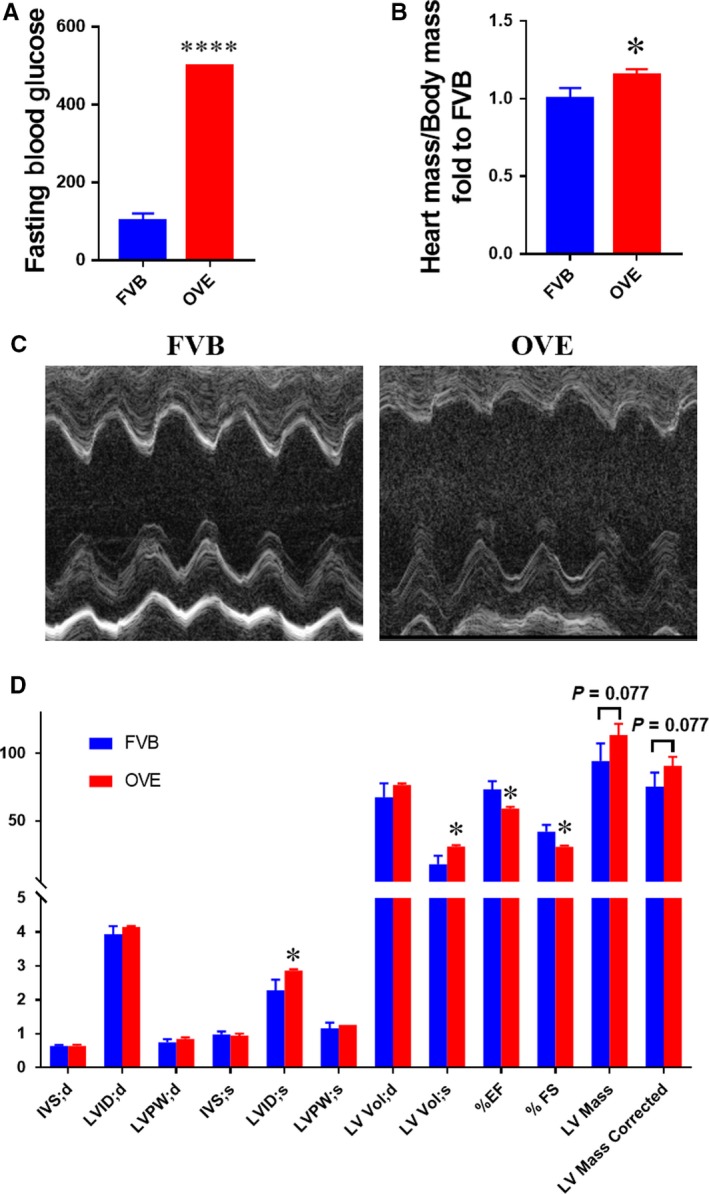
Cardiac structure and function in type 1 diabetic mice. OVE mice were used to establish a model of type 1 diabetes. A, Fasting blood glucose immediately prior to the killing. B, Heart mass‐to‐body mass ratio. C,D, Cardiac function, assessed using echocardiography. Data are presented as mean ± SD (n = 3 or 4). *P < 0.05 vs control FVB mice; ****P < 0.0001 vs control FVB mice

Peripheral blood glucose concentration was measured in whole blood from a tail vein prior to the killing, and diabetes was defined by a blood glucose >250 mg/dL. The blood glucose concentrations in the OVE group all exceeded the upper limit of the blood glucose meter, confirming the presence of diabetes (Figure 1A). Compared with the FVB group, the fasting blood glucose and heart mass/body mass ratio of the OVE group were significantly higher (Figure 1B).

Echocardiography showed that, compared with the FVB group, the left ventricular internal systolic diameter (LVID;s) and left ventricular systolic volume (LV Vol;s) were significantly higher in the OVE group, while the ejection fraction (EF) and fractional shortening (FS) were significantly lower than in FVB mice (Figure 1C,D). This indicates that T1D is associated with changes in the cardiac structure and cardiac dysfunction in OVE mice. The LV mass in the OVE mice tended to be higher, which is consistent with the trend in heart mass/body mass ratio, and suggests the presence of cardiac hypertrophy.

**Figure 2:**
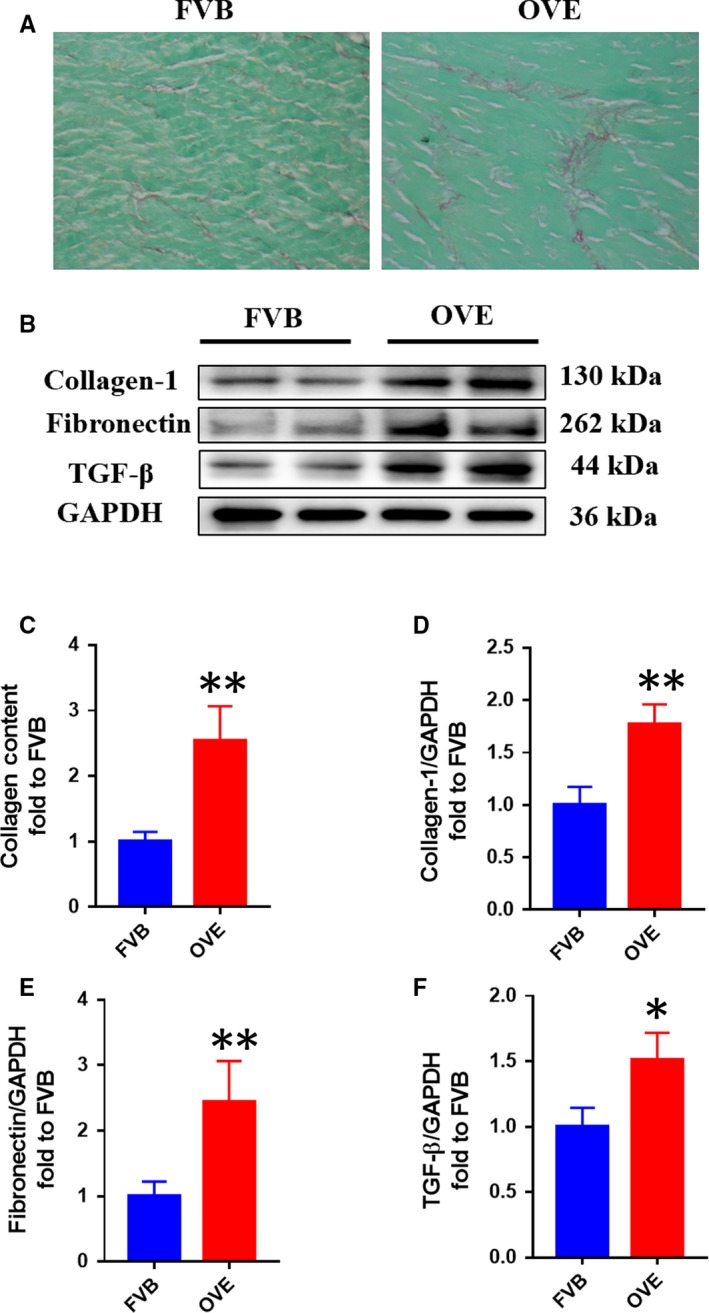
Cardiac fibrosis is significantly more severe in OVE mice than controls. A,C, Cardiac collagen deposition was assessed following Sirius Red staining. B,D‐F, Cardiac fibrosis was also assessed by Western blotting for fibronectin, collagen‐1 and growth factor beta. Data are presented as mean ± SD (n = 3 or 4). *P < 0.05 vs control FVB mice; **P < 0.01 vs control FVB mice

Using Sirius Red staining, we found that collagen deposition in the OVE group was significantly higher than in the FVB group (Figure 2A,C). Furthermore, the expression levels of fibronectin, collagen‐1 and TGF‐β in OVE mice were significantly higher by Western blotting (Figure 2B,D‐F). These findings indicate that the cardiac changes in OVE mice are consistent with the changes present in DCM.

### 3.2 Therapeutic effects of SFN and/or Zn in OVE mice

#### 3.2.1 General effects

**Figure 3:**
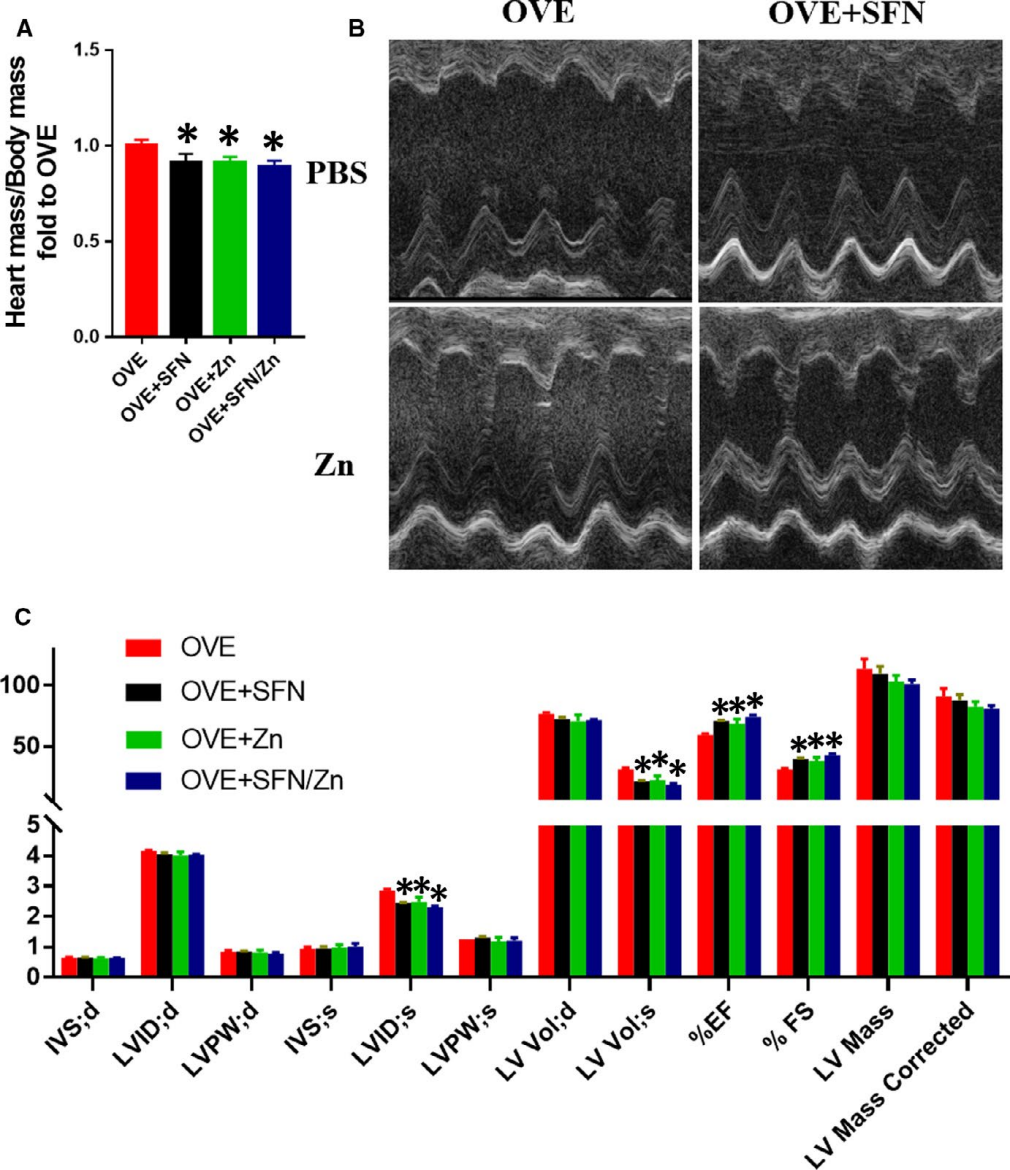
Sulforaphane (SFN) and/or zinc (Zn) treatment ameliorates type 1 diabetes‐associated defects in cardiac structure and function. Mice were administered SFN and/or Zn by gavage at doses of 0.5 and 5 mg/kg, respectively, 5 d a week for 18 wk, from 5 wk of age. Vehicle control mice were administered an equivalent volume of phosphate‐buffered saline containing 1% dimethyl sulphoxide. A, Heart mass‐to‐body mass ratio. B,C, Cardiac function, assessed using echocardiography. Data are presented as mean ± SD (n = 3 or 4). *P < 0.05 vs OVE mice

The blood glucose concentrations of the treatment groups also all exceeded the maximum measurable value of the blood glucose meter; therefore, there were no statistical differences in fasting blood glucose between the OVE group and the treatment groups. This indicates that SFN and Zn treatments have no noticeable effect on fasting blood glucose, which is consistent with previous studies.^46^, ^71^ Therefore, their cardioprotective effects are not achieved by lowering blood glucose levels. However, the heart mass/body mass ratio was significantly lower in the SFN‐treated group, the Zn‐treated group and the combined treatment group, with the combined treatment group showing a trend towards being even lower than in the other groups, although this difference did not reach statistical significance (Figure 3A).

#### 3.2.2 Cardiac structure and function

Compared with the OVE group, regardless of whether SFN, Zn or a combination was administered, LVID;s and LV Vol;s were significantly lower, while EF and FS were significantly higher than in controls. LV mass also tended to be lower in the treated groups, particularly in the combination treatment group (Figure 3B,C). Thus, the echocardiography data indicate that both monotherapy and the combination ameliorate T1D‐induced defects in cardiac structure and function.

#### 3.2.3 Treatment with SFN and/or Zn ameliorates myocardial fibrosis

**Figure 4:**
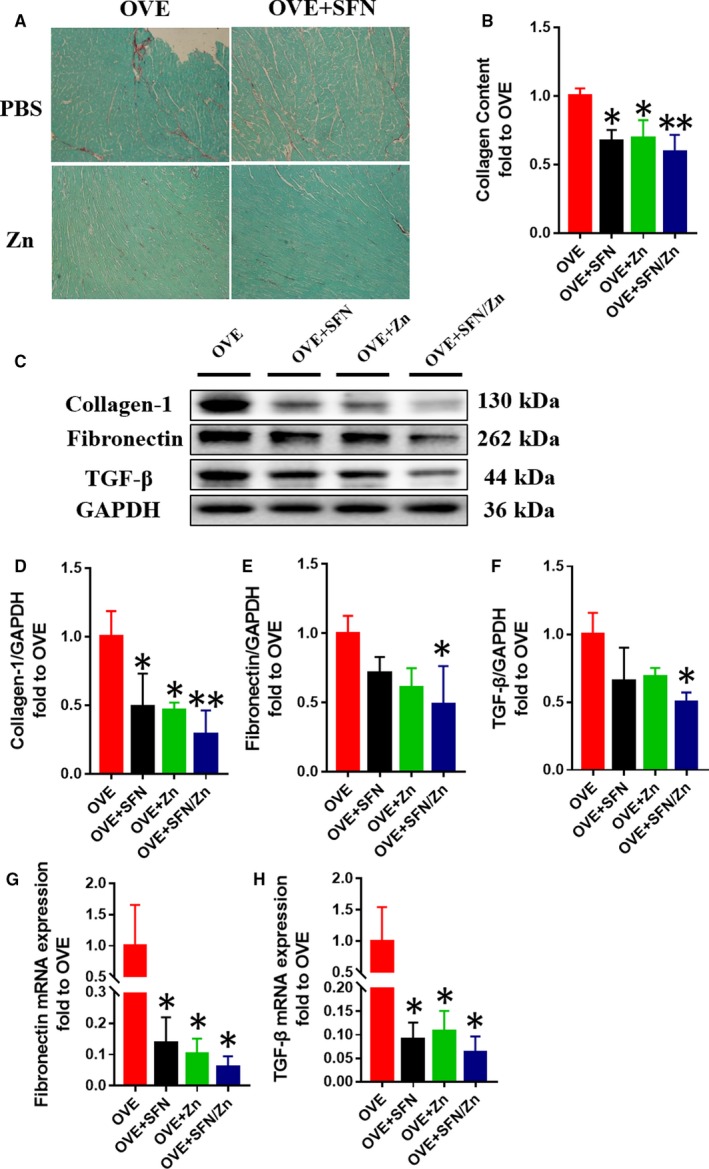
Sulforaphane and/or zinc treatment ameliorates cardiac fibrosis. A,B, Cardiac collagen deposition was assessed by Sirius Red staining. C‐F, Cardiac fibrosis was assessed by Western blotting for collagen‐1, fibronectin and transforming growth factor beta (TGF‐β). G,H, The mRNA expression of fibronectin and TGF‐β was measured by quantitative real‐time PCR. Data are presented as mean ± SD (n = 3 or 4). *P < 0.05 vs OVE mice; **P < 0.01 vs OVE mice

Fibrosis is another defect that is characteristic of DCM. Sirius Red staining showed that both the monotherapies and the combination treatment reduced myocardial collagen deposition in OVE mice, and the combined treatment group tended to have a greater effect, although this did not reach statistical significance (Figure 4A,B). Collagen‐1 protein expression was similarly reduced (Figure 4C,D). Furthermore, real‐time PCR analysis showed that all three treatments were able to ameliorate the T1D‐induced increases in fibronectin and TGF‐β mRNA levels, and the combined treatment group tended to have a larger effect (Figure 4G,H). Western blotting showed that fibronectin and TGF‐β protein levels tended to be lower in the monotherapy groups, but these differences did not reach statistical difference. However, the combination treatment did have a significant effect to reduce the expression of these proteins vs the OVE group (Figure 4C,E,F). These data demonstrate that treatment with SFN and/or Zn can ameliorate the ventricular remodelling, cardiac dysfunction and myocardial fibrosis associated with T1D.

### 3.3 Potential mechanism by which SFN and/or Zn treatment prevents T1D‐induced DCM in OVE mice

#### 3.3.1 Anti‐inflammatory and anti‐oxidative stress effects

**Figure 5:**
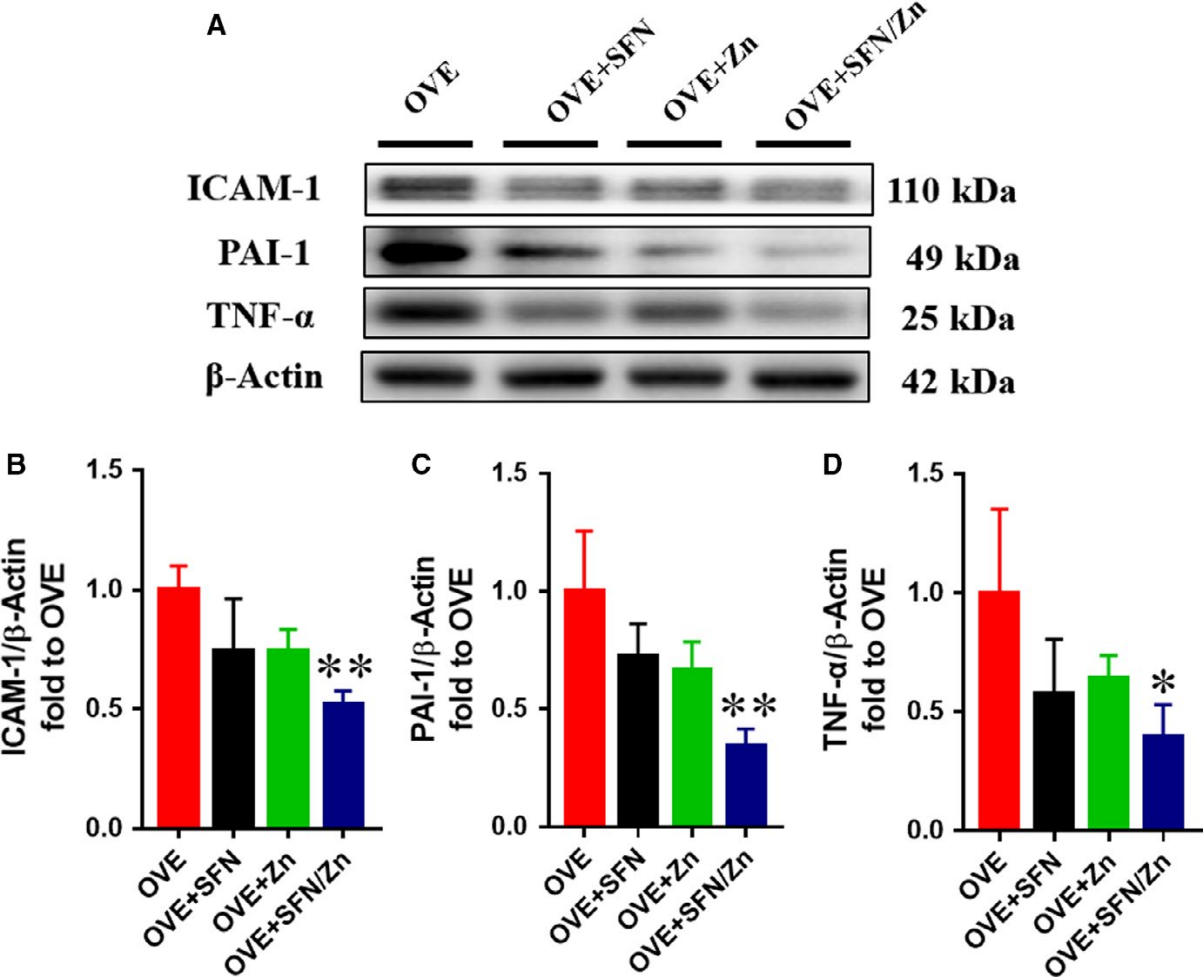
Sulforaphane and/or zinc treatment protects against the inflammation induced by type 1 diabetes. A‐D, Cardiac inflammation was assessed by Western blotting for intercellular adhesion molecule‐1, plasminogen activator inhibitor type 1, and tumour necrosis factor‐α. Data are presented as mean ± SD (n = 3 or 4). *P < 0.05 vs OVE mice; **P < 0.01 vs OVE mice

The combined treatment significantly reduced the protein expression of ICAM‐1, PAI‐1 and TNF‐α, while each monotherapy had weaker, non‐significant effects on the expression of these proteins (Figure 5). This shows that the combination of SFN and Zn can significantly ameliorate the myocardial inflammation induced by T1D.

**Figure 6:**
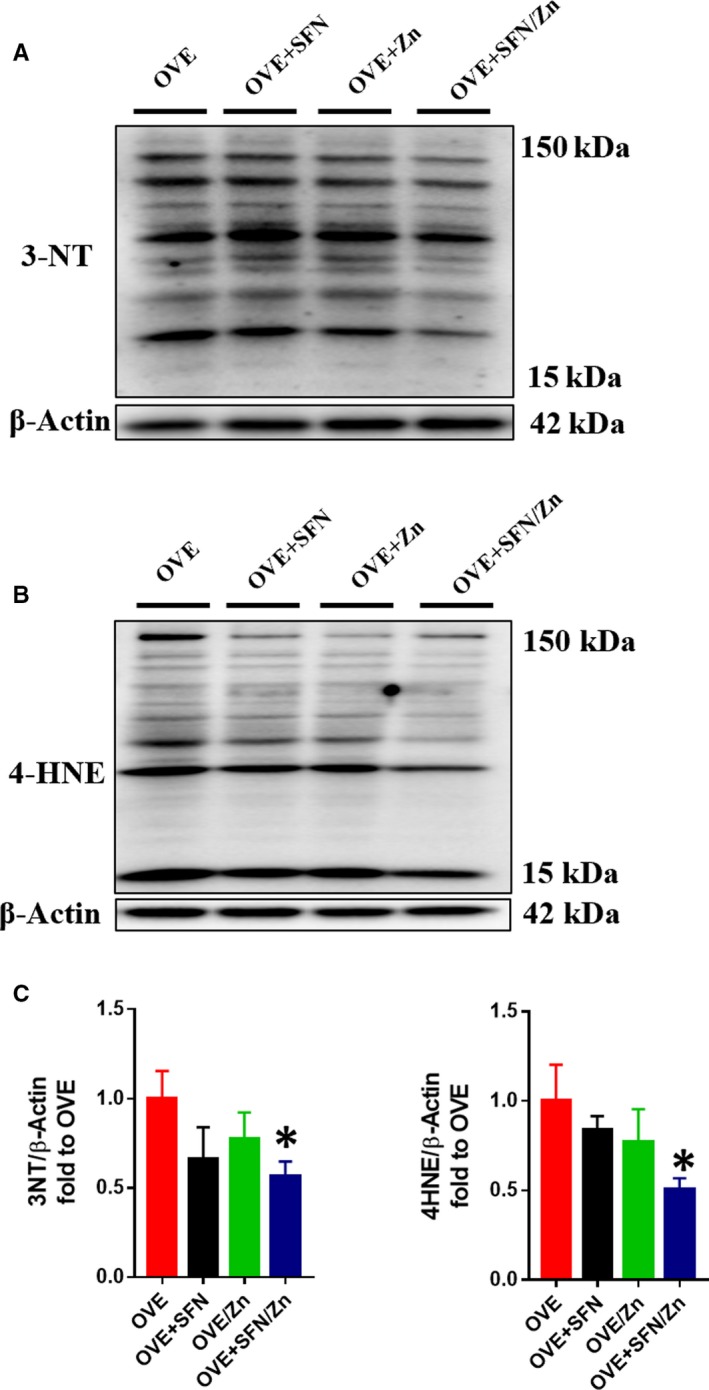
Sulforaphane and/or zinc treatment protects against oxidative stress induced by type 1 diabetes. A‐D, Cardiac oxidative damage was assessed using Western blot analyses of 3‐nitrotyrosine and 4‐hydroxynonenal. Data are presented as mean ± SD (n = 3 or 4). *P < 0.05 vs OVE mice

We used 3‐NT and 4‐HNE as indicators of the severity of oxidative stress. Similar to their effects on inflammatory markers, the monotherapies were able to reduce 3‐NT and 4‐HNE expression to varying degrees, but not significantly, whereas the combination treatment did significantly reduce the expression of these oxidative stress markers (Figure 6).

#### 3.3.2 Activation of Nrf2 and its downstream targets

**Figure 7:**
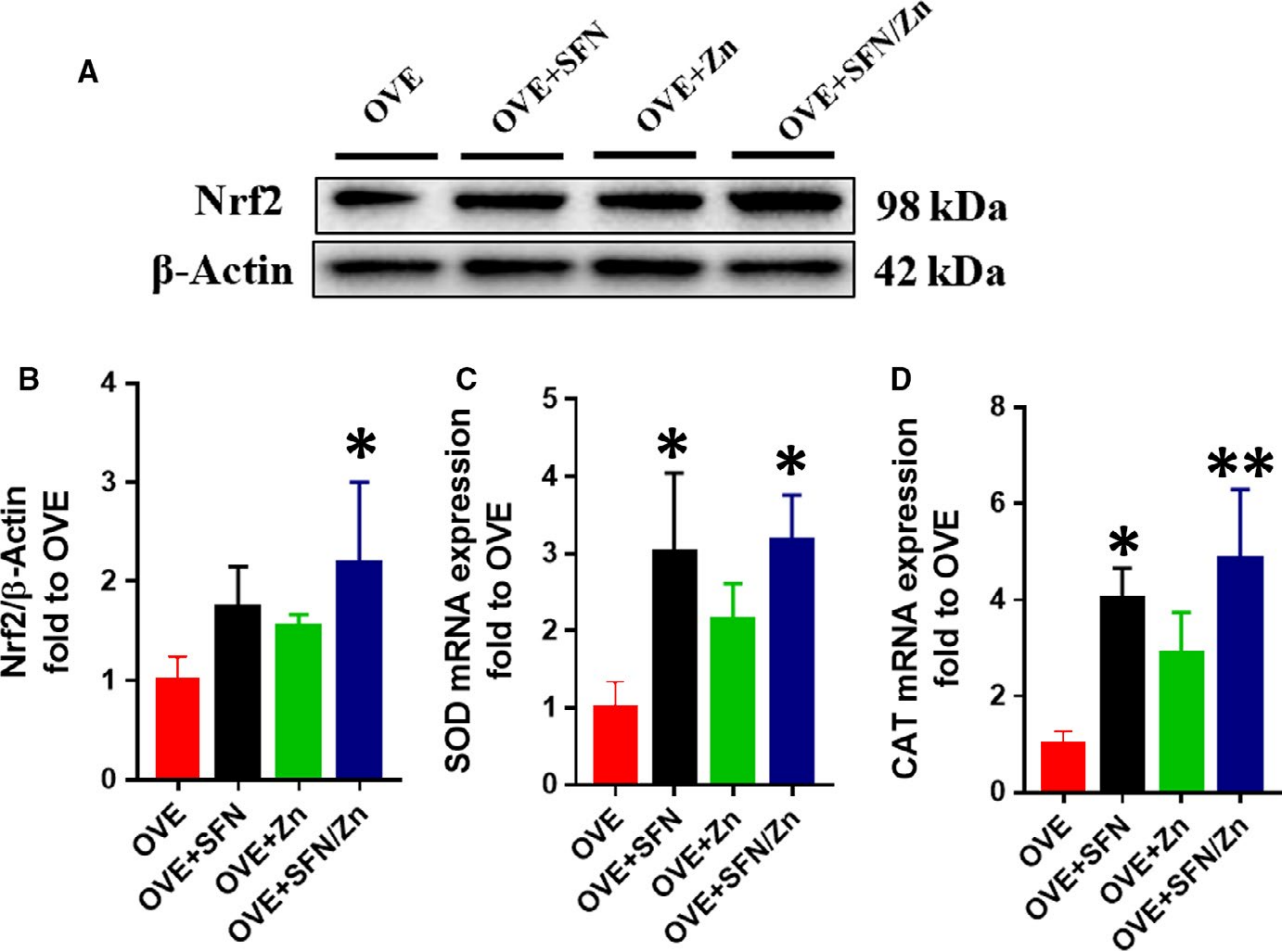
Sulforaphane and/or zinc treatment increases the protein expression of nuclear factor E2–related factor 2 (Nrf2) and mRNA expression of its downstream genes. A,B, The expression level of Nrf2 protein was measured by Western blotting. C,D, Nrf2 activation was assessed by measuring the mRNA expression of downstream target genes of Nrf2, superoxide dismutase‐2 and catalase, by quantitative real‐time PCR analysis. Data are presented as mean ± SD (n = 3 or 4). *P < 0.05 vs OVE mice; **P < 0.01 vs OVE mice

**Figure 8:**
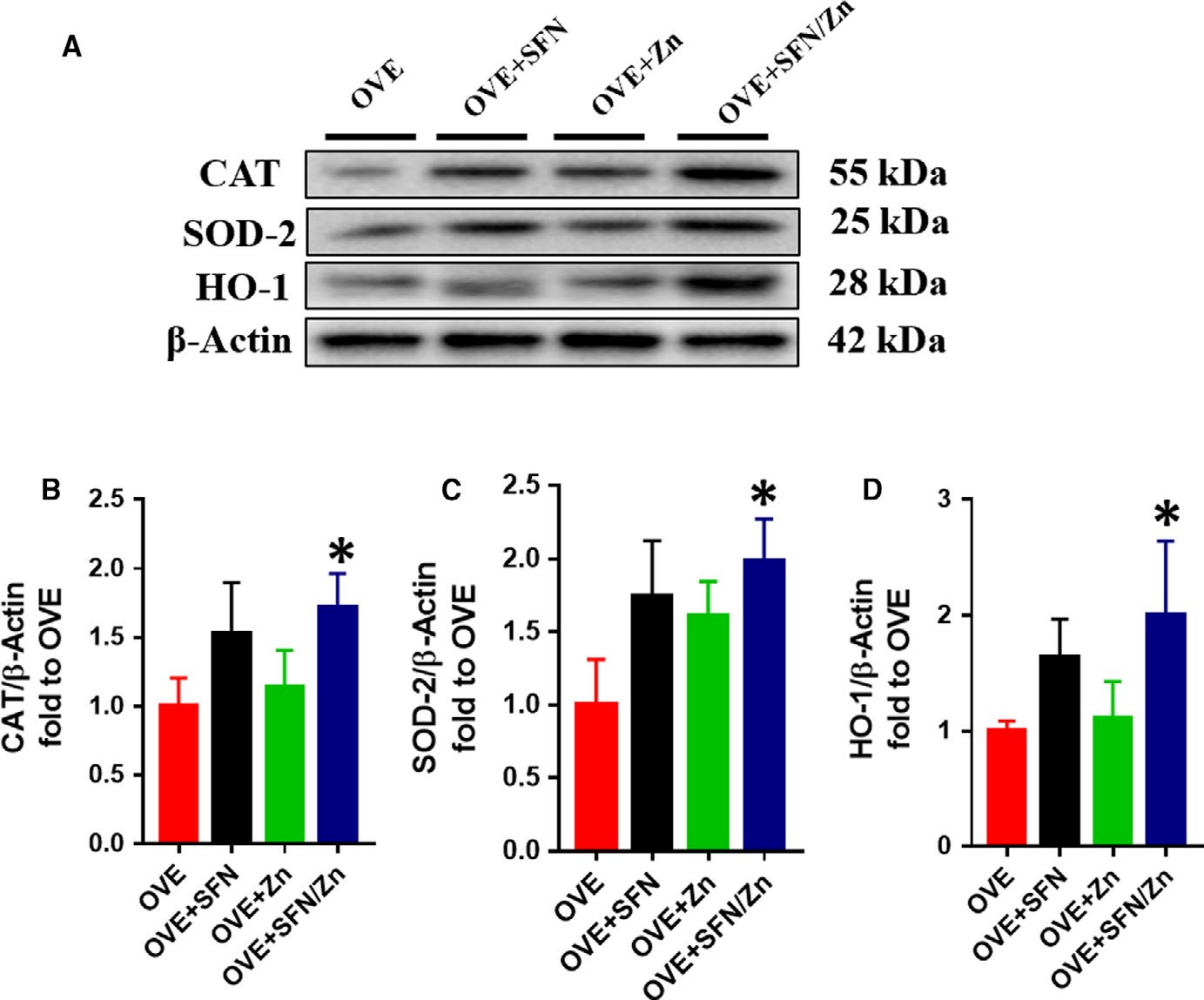
Sulforaphane and/or zinc treatment increases protein expression of nuclear factor E2–related factor 2 (Nrf2) downstream targets. A‐D, Protein expression of CAT, SOD‐2 and haem oxygenase‐1 (HO‐1), measured using Western blotting. The expression of CAT, SOD‐2 and HO‐1 protein was determined using the same membrane as that used to determine the expression of Nrf2 proteins in Figure 7A. The β‐actin membrane was the same as that used in Figure 7. Data are presented as mean ± SD (n = 3 or 4). *P < 0.05 vs OVE mice

Because Nrf2 and its downstream targets play an important antioxidant role, we measured total Nrf2 expression, which was slightly higher in both the SFN and Zn groups, and significantly higher in the combination treatment group (Figure 7A,B). However, because greater expression of Nrf2 does not necessarily imply greater activity, we evaluated its transcriptional activity by measuring mRNA expression of its downstream targets, the antioxidants SOD (Figure 7C) and CAT (Figure 7D). Numerous studies have shown that Nrf2 downstream targets, such as SOD and CAT, have significant antioxidant effects.^72^, ^73^, ^74^, ^75^ The mRNA levels of both CAT and SOD in the SFN and combination groups were significantly higher than those in the OVE group, and also slightly higher than those in the Zn group (Figure 7C,D). We also measured the expression of CAT and SOD, as well as HO‐1, at the protein level. In the combination treatment group, the expression of CAT, SOD‐2 and HO‐1 was significantly higher than in the OVE group, but although expression also tended to be higher in the SFN‐ and Zn‐treated groups, it was not significantly different (Figure 8). These results suggest that SFN is more effective than Zn at activating Nrf2, although Zn has an additive effect.

#### 3.3.3 Up‐regulation of MT expression

**Figure 9:**
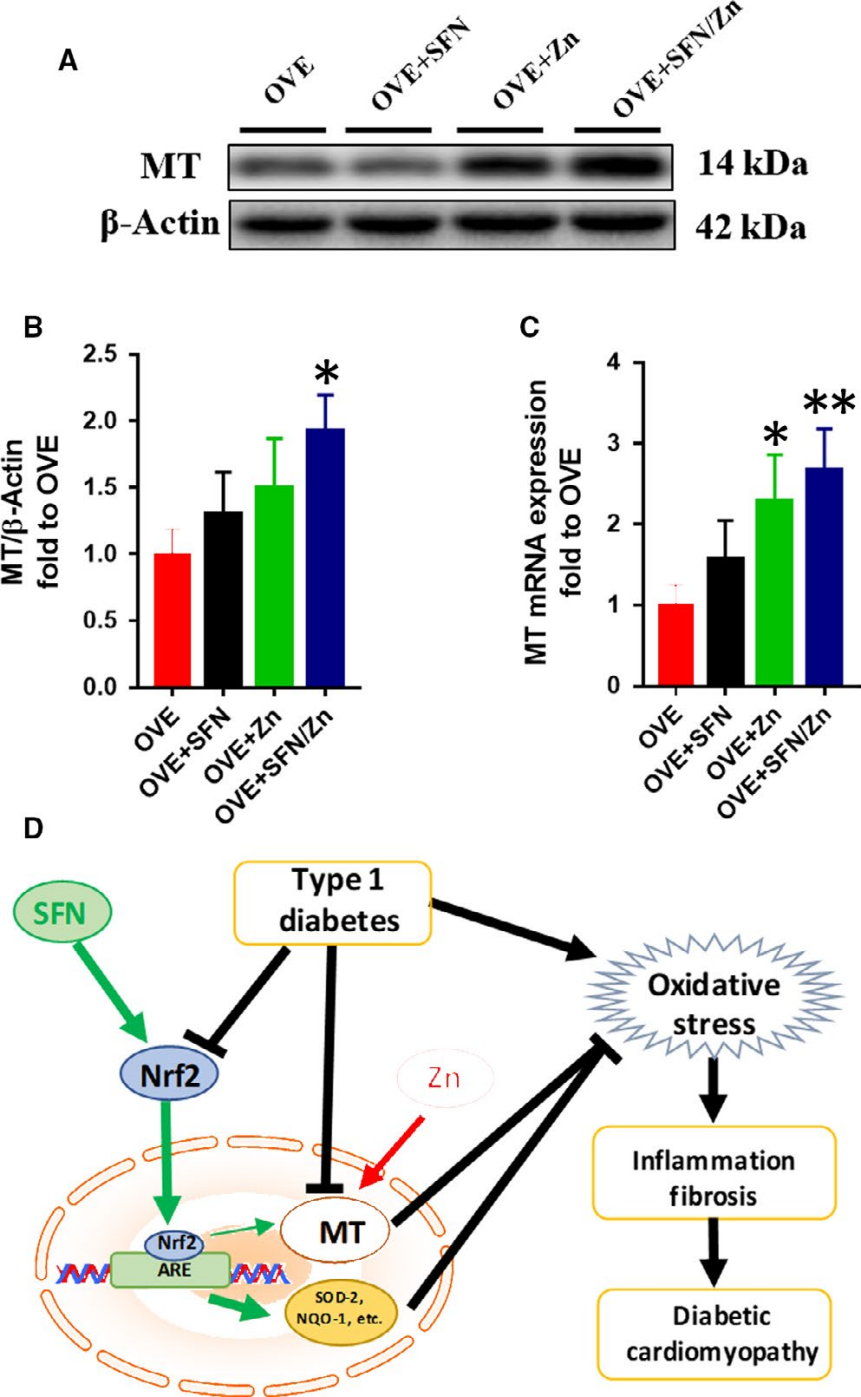
Sulforaphane and/or zinc treatment increases mRNA and protein expression of metallothionein (MT). A,B, Protein expression of MT measured using Western blotting. C, mRNA expression of MT measured using quantitative real‐time PCR. D, Schematic illustration of the effects of SFN and/or Zn treatment on diabetic cardiomyopathy induced by T1D. SFN activates Nrf2 and its downstream genes, and MT can be also up‐regulated by SFN via Nrf2. Zn directly activates MT, and the activation of these signalling pathways by SFN and Zn has additive effects on anti‐oxidative stress. Data are presented as mean ± SD (n = 3 or 4). *P < 0.05 vs OVE mice; **P < 0.01 vs OVE mice

Expression of MT was also measured in each group at both the protein (Figure 9A,B) and mRNA (Figure 9C) levels. In the combination treatment group, MT protein expression was significantly higher than in the OVE group and slightly higher than in both SFN and Zn groups. Specifically, the mRNA expression of MT was significantly higher in Zn‐treated and SFN/Zn‐treated OVE mice than in the OVE group. These findings suggest that MT gene and protein expression respond more to Zn treatment than SFN treatment.

## 4 DISCUSSION

Previous studies have clearly shown that both Nrf2 and MT play important roles in the prevention of DCM.^26^, ^29^, ^46^ For the induction of Nrf2, most studies used SFN,^58^, ^76^, ^77^ whereas studies on the induction of MT used Zn.^78^, ^79^, ^80^ To date, no studies have evaluated the combination of SFN and Zn for the treatment of DCM. Here, for the first time, we have used these inducers of Nrf2 and MT in combination, and have shown that: (a) treatment with SFN and/or Zn can effectively prevent DCM in OVE mice; (b) Nrf2 and its downstream targets play an important role in the effects of SFN and Zn; and (c) compared with the use of SFN or Zn alone, use of the combination delivered more significant cardioprotective effects, with the two substances having additive effects.

Our previous studies have shown that in WT mice SFN can induce MT expression, but this does not occur in Nrf2‐KO mice; however, the absence of MT does not affect the induction of Nrf2 and its classical downstream targets by SFN.^45^, ^46^ This indicates that MT is downstream of Nrf2. In addition, Zn can induce MT expression; therefore, a combination of the two substances can activate MT more effectively. In our study, the expression of MT in the combined treatment group was the highest.

We have also found that MT‐KO mice are more susceptible to oxidative stress in disease states, resulting in a further decline in Nrf2 compared with WT mice. In mice with high MT expression, Nrf2 was significantly higher than in WT mice,^47^ suggesting that MT also regulates the expression of Nrf2. In another study, we found that Zn stimulates Nrf2 expression and transcription via activation of Akt‐dependent inhibition of Fyn nuclear translocation.^48^ Fyn is a negative regulator of Nrf2 because it enters nuclei and exports Nrf2 to the cytosol, where it is degraded.^81^ The translocation of Fyn into the nucleus is mediated by glycogen synthase kinase (GSK)‐3β, which is active in its dephosphorylated form and inactive when phosphorylated.^82^ The use of Zn to induce MT preserves cardiac Akt2 signals by inhibiting tribbles homolog 3 (TRB3)^83^; consequently, GSK‐3β phosphorylation may result in a reduction in the nuclear translocation of Fyn and the export of Nrf2 to the cytosol. In this study, we have shown that the expression of Nrf2 and its classical downstream targets was also highest in the combination treatment group.

The protective effects of SFN are completely dependent on Nrf2, and the expression of genes such as NQO‐1 and HO‐1 is dependent on that of Nrf2.^45^, ^46^ However, not only Nrf2 is the transcription factor that activates MT transcription, but also other transcription factors, such as metal‐responsive element‐binding transcription factor‐1 (MTF‐1), may regulate MT transcription.^84^ Nrf2 or MT gene mutations sometimes occur,^85^, ^86^ and when Nrf2 mutations are present, the Nrf2 signalling pathway is less effective at protecting against diabetes‐associated myocardial damage, but in this situation, the MT signalling pathway can compensate. Conversely, when an MT mutation causes the MT signalling pathway to be less effective, the Nrf2 signalling pathway can compensate. This is consistent with our finding that the use of a combination of SFN and Zn is more effective in the prevention of DCM.

Sulforaphane has been frequently used in combination with other drugs to treat diseases. SFN and 5‐fluorouracil have been shown to synergistically reduce cell growth in a triple‐negative breast cancer line by inducing autophagic cell death and premature senescence.^87^ In addition, epidermal squamous cell carcinoma and malignant mesothelioma can be treated using a combination of SFN and cisplatin, which inhibits tumour cell proliferation and accelerates tumour cell autophagy.^88^, ^89^ Furthermore, the combination of SFN and myricetin can induce fat cell apoptosis through Akt‐mediated mitochondrial apoptosis,^90^ which may also represent a novel strategy for the treatment of obesity. Finally, in inflammatory bowel disease, a combination of SFN and selenium synergistically up‐regulates TrxR‐1, which plays an important role in maintaining intracellular redox homeostasis and contributes to the SFN‐induced protection against free radical–mediated oxidative damage in normal colon cells.^91^

Zinc also has many advantages when used in combination with other drugs to treat diseases. For example, a combination of Zn and trans‐retinoic acid is more effective at inhibiting the growth of *Listeria monocytogenes*.^92^ Another study demonstrated that a combination of Zn and glibenclamide limits cardiovascular complications in diabetic rats.^93^ Thus, combination therapy not only permits beneficial drug interactions, but also can reduce the adverse effects of other drugs, while achieving better therapeutic results.

In our study, many parameters were significantly different between the combination group and the OVE group, but this was not often the case with the monotherapy groups. The reasons for this are also worth discussing. Our experiments demonstrated therapeutic, rather than prophylactic, effects because treatment began after diabetes had been established in the mice. At the time of drug administration, the mice are likely to already have diabetic myocardial damage, which will influence the efficacy of the drug. In addition, SFN was used at a dose of 0.5 mg/kg, which is a relatively low dose, which may also affect the efficacy of the treatment. In future experiments, we plan to use higher drug doses, and possibly transgenic mice, to better define the therapeutic effects of SFN and Zn against DCM.

The present study has several limitations: (a) the number of animals per group was low because of the low reproductive rate of OVE mice and (b) we did not compare FVB and its treatment groups with OVE and its treatment groups because comparisons between eight groups of mice would have been complicated to perform.

In summary, our study is the first to use SFN and Zn in combination and to observe their combined efficacy against DCM in OVE mice. By effectively activating Nrf2 and its classical downstream targets, and MT, this combination can ameliorate the defects associated with DCM more effectively than monotherapy. These findings may suggest new avenues for the future clinical treatment of DCM.

## CONFLICT OF INTEREST

The authors confirm that there are no conflicts of interest.

## AUTHOR CONTRIBUTIONS

QL and LC originally designed the project. JW performed experiments, analysed data and wrote the manuscript. SW, WW, JC, QZ and ZZ performed partial experiments and data collection. LC was responsible for scientific review and manuscript editing. QL and LC monitored project progression, modified the experimental designs and revised the manuscript. All authors approved the final version of the manuscript.
